# Real-world evidence of tezepelumab for severe asthma: a retrospective multicentre cohort

**DOI:** 10.1183/23120541.00314-2025

**Published:** 2025-09-01

**Authors:** Jasmin Khateeb, Mordechai R. Kramer, Ophir Freund, Reem Mhameed, Eviatar Naamany, Aviv Kupershmidt, Gal Elkayam, Anna Breslavsky, Dror Rosengarten, Yaniv Dotan, Ori Wand, Amir Bar-Shai

**Affiliations:** 1 Pulmonary Institute, Rambam Health Care Campus, Haifa, Israel; 2 Ruth & Bruce Rappaport Faculty of Medicine, Technion – Israel Institute of Technology, Haifa, Israel; 3 Translational Lung Lab, Clinical Research Institute at Rambam, Rambam Health Care Campus, Haifa, Israel; 4 Department of Pulmonology, Rabin Medical Center, Petah Tikva, Israel; 5 Faculty of Medicine, Tel Aviv University, Tel-Aviv, Israel; 6 Institute of Pulmonary Medicine, Tel Aviv Sourasky Medical Center, Tel-Aviv, Israel; 7 Division of Pulmonary Medicine, Barzilai University Medical Center, Faculty of Health Sciences, Ben-Gurion University of the Negev, Beersheba, Israel

## Abstract

**Background:**

Tezepelumab, a monoclonal antibody targeting thymic stromal lymphopoietin, has demonstrated efficacy for severe asthma in clinical trials, but real-world evidence remains limited. We aimed to evaluate the characteristics and outcomes of patients initiating tezepelumab in a real-world setting.

**Methods:**

We conducted a retrospective, multicentre cohort study across four tertiary care centres to evaluate the real-world effectiveness of tezepelumab in patients with severe asthma. Eligible patients were adults with a confirmed diagnosis of severe asthma, treated with tezepelumab. Data on exacerbation rates, pulmonary function and corticosteroid use were collected and analysed at baseline and 1-year follow-up.

**Results:**

The study included 103 patients treated with tezepelumab with a median (interquartile range) duration of 323 (267–359) days. Overall, 39% had prior biologic therapy and 32% had an eosinophil count <300 cells·μL^−1^. At follow-up, there was a 66.7% relative reduction in annual exacerbations. The most pronounced reduction was observed in biologic-naïve patients with peripheral eosinophil counts ≥300 cells·μL^−1^ (78% reduction). A 62% relative reduction was found among patients with eosinophil counts <150 cells·μL^−1^. There were significant improvements in forced expiratory volume in 1 s, with 51% of patients demonstrating a ≥10% relative increase. Of patients using maintenance oral corticosteroid (mOCS), 45% discontinued mOCS and 23% reduced their dose by more than 50%.

**Conclusions:**

In this real-world cohort, treatment with tezepelumab for a median of 46 weeks was associated with improved asthma control, reductions in exacerbations, mOCS and inhaled corticosteroid doses, and a low symptom burden. These findings were consistent in biologic-experienced or low-eosinophil patients.

## Shareable abstract

**Tezepelumab initiation was associated with robust improvements in severe asthma within a multicentre real-world cohort. It shows potential as a first-line treatment for the different asthma phenotypes, as well as following other biologic treatment failure.** https://bit.ly/4hVQusV

## Introduction

Asthma is a complex, chronic disease characterised by heterogeneous airway inflammation and airway hyperresponsiveness (AHR). It is considered one of the leading chronic illnesses, with increasing prevalence and a significant global health and financial burden [1]. Severe asthma, which is defined as uncontrolled or exclusively controlled with high-dose inhaled corticosteroids (ICS) plus a second controller, is a subset of asthma accounting for approximately 5–10% of all asthma cases worldwide and is responsible for the most healthcare expenditure, greatest morbidity and highest risk for death compared to nonsevere asthma [2]. Tezepelumab, a human monoclonal antibody that specifically targets thymic stromal lymphopoietin (TSLP), an epithelial cytokine that is implicated in the initiation and persistence of asthma airway inflammation, is a relatively newly approved biologic addition in the field of severe asthma management and is used in conjunction with other maintenance treatment of severe asthma.

A variety of inflammatory pathways are activated in patients with severe asthma, mainly type 2 inflammation such as IgE-mediated mast cell and eosinophilic activation pathways, and is chiefly orchestrated by interleukins (IL)-4, IL-5 and IL-13, with TSLP as an upstream molecule, resulting in airway epithelial dysregulation, airway hyperreactivity and fibroblast proliferation, eventually leading to airway remodelling and obstruction [3]. Prior to the widespread use of biologics, long-term reliance on systemic steroids was the mainstay of severe asthma treatment, with a high burden and costs from many complications including metabolic syndrome, cardiovascular events, psychiatric disorders, osteoporosis and chronic immune suppression [4, 5]. A revolutionary development in severe asthma treatment occurred with the development of targeted biological therapies that specifically address type 2 inflammatory pathways in asthma. They have been shown to be highly effective for asthma control, reducing the need for systemic corticosteroids and improving quality of life [6]. This was the case for tezepelumab as well, which also showed efficacy in patients with low type-2 inflammation [7].

Although randomised controlled trials (RCTs) established the significant efficacy of tezepelumab in improving both airway inflammatory markers and clinical parameters in asthma control, including a reduction in exacerbations, while maintaining an excellent safety profile, long-term outcomes and diverse patient population data are insufficient [8–10]. In fact, many severe asthma patients were excluded due to strict selection protocols and those included were highly adherent and more closely monitored compared to real-world populations, highlighting the need for external validity and real-world data to support the findings of these RCTs [11, 12].

In this study, we aimed to assess the efficacy of biological treatment with tezepelumab in a real-world population of individuals with severe asthma. By using data from a broad and heterogenous population, including biologic-naïve and biologic-experienced patients, as well as those with high or low eosinophil counts, reflecting the population heterogeneity of severe asthma with various comorbidities, we aim to assess the performance of tezepelumab beyond the confines of clinical trial protocols.

## Methods

### Study design

This is a retrospective observational multicentre study that enrolled patients from four tertiary care centres located in the south, centre and north of Israel, treated with tezepelumab for severe asthma. Eligible patients for the study inclusion were adults (≥18 years) diagnosed with severe asthma according to European Respiratory Society/American Thoracic Society definition, that are receiving or received tezepelumab per routine clinical practice, *i.e.* 210 mg every 4 weeks for severe asthma.

In general, tezepelumab has been reimbursed in Israel for severe asthma since early 2023. Criteria for prescribing initial biological therapies in Israel include uncontrolled asthma resulting in three or more exacerbations that required oral corticosteroids (OCS) in the previous year or requiring maintenance OCS (mOCS) at a cumulative annual dose exceeding an equivalent of 900 mg of prednisone, despite stable treatment with a high-dose inhaled corticosteroid (ICS) long-acting beta-agonist.

The study adheres with the principles of the Declaration of Helsinki and Good Clinical Practice guidelines and was approved by the institutional review board (TLV-0106-24), with a waiver from informed consent based on its retrospective design. Results are reported according to STROBE (STrengthening the Reporting of OBservational studies in Epidemiology) statement guidelines.

### Patient data and data analysis

Patient data were collected from electronic health records during routine clinic visits and follow-ups between February 2023 and February 2025. We focused on 1-year outcomes, using the most adjacent follow-up data for each patient. Patients with at least 6 months follow-up were included in our analysis.

Data were collected and entered into a standardised database in each centre. Baseline data refers to data prior to treatment initiation and included patient characteristics, exacerbation rate, maintenance treatment, blood tests and pulmonary function tests. Exacerbation rates and mOCS were averaged from a similar time duration as the duration of follow-up per patient. ICS dose was divided into groups based on budesonide equivalent doses (high: >800 µg·day^−1^; medium: 400–800 µg·day^−1^). Asthma control test (ACT) results were collected from patients' follow-up notes. This test was incorporated into routine care during 2024 in three of the four included centres; hence, it was only available in the post-treatment evaluation.

The cohort was divided into three groups, based on prior real-world studies, as follows [13]: 1) biologic-experienced (patients who received a biological therapy for severe asthma during the pre-treatment period); 2) biologic-naïve with peripheral eosinophil counts ≥300 cells·μL^−1^; and c) biologic-naïve with peripheral eosinophil count of<300 cells·μL^−1^. An additional analysis of exacerbation effects was conducted for patients with eosinophils <150 cells·μL^−1^ based on the main phase-3 tezepelumab study [7].

The highest value of peripheral blood eosinophil in the 1 year prior to treatment was used in our analysis. Pulmonary function measures included forced expiratory volume in 1 s (FEV_1_), forced vital capacity (FVC), FEV_1_/FVC ratio and forced expiratory flow at 25–75% of FVC (FEF_25–75%_). As part of routine follow-up in most centres for patients receiving biologics, spirometry is performed without bronchodilators; therefore, only pre-bronchodilator values were included. Asthma exacerbations were defined as those leading to OCS use for ≥3 days, an emergency department visit or hospitalisation.

The study outcomes included annual exacerbation rate (AER), total number of exacerbations, mOCS use and dose, ICS dose, and change in FEV_1_ % predicted. The AER was calculated with the following formula: (total exacerbations/total duration of follow-up (days))×365. Change in FEV_1_ % pred was calculated with the following: follow-up % FEV_1_–baseline % FEV_1_. Relative change in FEV_1_ was calculated with the following: change in FEV_1_ % pred/baseline FEV_1_ % pred. We also assessed a combined outcomes measure that included no exacerbations following therapy initiation, an improvement of FEV_1_ (defined as relative change ≥0.1) and cessation of mOCS use for individuals who had required such therapy.

### Statistical analysis

Categorical variables were expressed as frequency and percentages and compared between groups by Chi-square tests. The distribution of continuous variables was assessed using histograms and the Kolmogorov–Smirnov test with all continuous variables showing a non-normal distribution. Continuous variables were described using medians and interquartile ranges (IQRs) and compared between groups using Mann–Whitney U tests. Pre–post comparisons of FEV_1_ were conducted using the paired-samples Wilcoxon signed-rank test. Predictors for combinations of the different outcomes were evaluated using univariate analysis with the tests mentioned above, including calculation of odds ratios and 95% confidence intervals. All analyses were performed with IBM SPSS 27.0 software (SPSS Inc., Chicago, IL).

## Results

**FIGURE 1:**
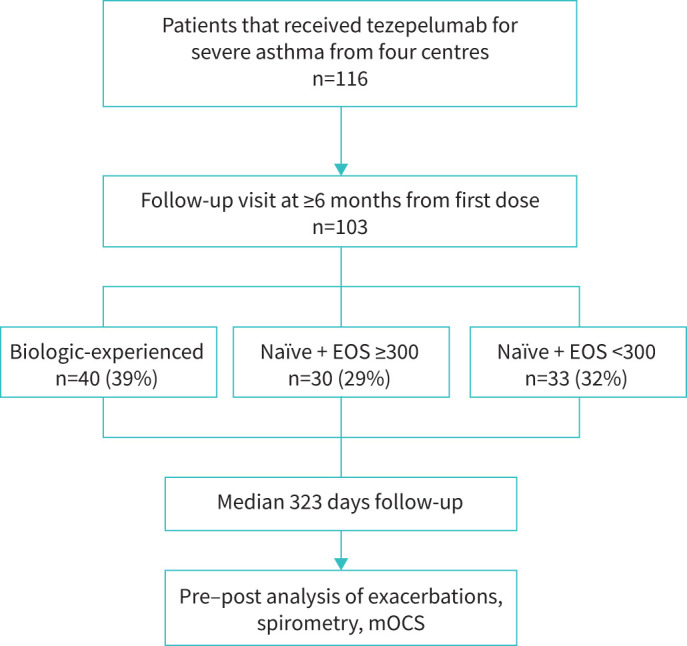
Flowchart of the study inclusion process. EOS: eosinophils; mOCS: maintenance oral corticosteroids.

Overall, 116 patients with a confirmed diagnosis of severe asthma initiated tezepelumab at one of the four centres. After the exclusion of 13 patients who were lost to follow-up, 103 patients who received at least 6 months of treatment were included in this study (figure 1). Baseline demographics and clinical characteristics are shown in table 1.

**TABLE 1:** Study population characteristics

Variable	All patients n=103 (%)	Naïve, EOS <300 n=33 (%)	Naïve, EOS ≥300 n=30 (%)	Biologic-experienced n=40 (%)	p-value
**Age, median (IQR)**	56 (42–69)	56 (45–69)	58 (42–73)	57 (43–69)	0.914
**Female sex**	68 (66)	22 (67)	18 (60)	28 (70)	0.679
**Smoking**					0.130
Past	16 (16)	4 (12)	6 (21)	6 (15)	
Current	12 (12)	4 (12)	0 (0)	8 (20)	
**Chronic rhinosinusitis**	15 (15)	3 (9)	4 (13)	8 (20)	0.447
**Nasal polyposis**	14 (12)	4 (13)	3 (9)	6 (13)	0.634
**Bronchiectasis**	15 (13)	4 (12)	5 (17)	6 (15)	0.873
**Metabolic comorbidity^#^**	31 (30)	11 (33)	9 (30)	11 (28)	0.864
**FEV_1_, % pred, median (IQR)**	65 (49–82)	62 (49–79)	69 (58–93)	64 (48–75)	0.283
**FEV_1_, litres, median (IQR)^¶^**	1.69 (1.22–2.04)	1.63 (1.23–2.02)	1.76 (1.36–2.21)	1.63 (1.16–2.04)	0.765
**FVC, % pred, median (IQR)**	75 (60–91)	72 (56–88)	86 (69–95)	71 (56–86)	0.068
**FEV_1_/FVC, median (IQR)**	0.73 (0.61–0.82)	0.68 (0.61–0.79)	0.73 (0.58–0.80)	0.75 (0.62–0.83)	0.347
**FEF_25–75%_, % pred, median (IQR)^¶^**	48% (26–79)	45% (24–70)	53% (29–74)	47% (32–89)	0.426
**Eosinophils, cells·μL^−1^, median (IQR)**	200 (100–400)	100 (100–200)	400 (300–500)	200 (100–400)	<0.001
**IgE, IU·mL^−1^, median (IQR)^¶^**	157 (58–330)	93 (54–285)	150 (79–280)	191 (71–531)	0.243
**Maintenance inhaled corticosteroids**					0.691
Moderate dose	36 (35)	12 (36)	9 (30)	15 (37)	
High dose	66 (64)	21 (64)	20 (67)	25 (63)	
**LAMA**	43 (42)	9 (27)	11 (37)	23 (58)	0.027
**Maintenance OCS**	40 (39)	11 (33)	9 (30)	2 (50)	0.173

EOS: eosinophils; FEF_25–75%_: forced mid-expiratory flow; FEV_1_: forced expiratory volume in 1 s; FVC: forced vital capacity; IQR: interquartile range; LAMA: long-acting muscarinic antagonists; OCS: oral corticosteroids. ^#^: Including hyperlipidaemia, hypertension and diabetes. ^¶^: Data missing for FEV_1_ in litres (n=79), FEF_25–75%_ (n=86) and IgE levels (n=79).

The cohort's median (IQR) age was 56 (42–69) years, 66% were female and 28% were past or current smokers (>10 pack-years). 40 patients (39%) had received biological therapy in the 6 months prior to tezepelumab initiation, which included dupilumab (n=14), mepolizumab (n=10), benralizumab (n=12) and omalizumab (n=4). In addition, 18 patients (17%) had received more than one biologic therapy in the past. The median (IQR) baseline FEV_1_ was 65% pred (49–82%).

The overall median (IQR) duration from the first treatment to follow-up was 323 (267–359) days, *i.e.* 46 (38–51) weeks. 11 patients (11%) stopped tezepelumab during follow-up. Of those patients, eight stopped due to worsening respiratory symptoms and three due to conceived side-effects (post-administration bone pain, weakness or tremors). One patient died at 13 months after initiation (beyond included follow-up) following a severe urinary infection.

### Exacerbations

**FIGURE 2:**
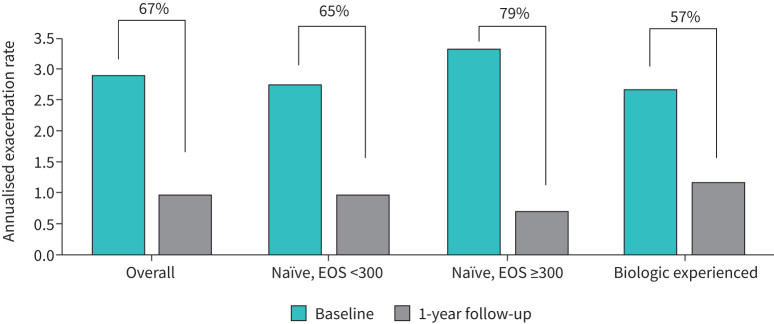
Exacerbation rates for patients 1 year before and after tezepelumab initiation, overall and by group, with the relative reduction percentage of exacerbations. EOS: eosinophils.

Exacerbation analyses at baseline and follow-up are shown in table 2 and figure 2. Overall, 4% had no exacerbations in the time period prior to treatment, compared to 43% at follow-up (p<0.01). The AER was 2.88 (95% CI 2.64–3.12) at baseline and 0.96 (95% CI 0.74–1.18) at follow-up (p<0.01), with a 66.7% relative reduction of exacerbations. Patients who were naïve to biologic therapy with eosinophil counts ≥300 cells·μL^−1^ had the highest relative reduction in exacerbations (79%), while those with prior biologic therapy had the lowest reduction (57%), although changes between groups were not statistically significant (p=0.10). In a sub-analysis including patients naïve to biologic therapy with eosinophil counts <150 cells·μL^−1^ (n=18), there was a relative reduction of 63% in exacerbations at follow-up. Of note, six of these patients (33%) received mOCS at baseline.

**TABLE 2:** Exacerbations before and after treatment with tezepelumab^#^

Variable	All patients n=103 (%)		Naïve, EOS <300 n=33 (%)		Naïve, EOS ≥300 n=30 (%)		Biologic-experienced n=40 (%)	
	Pre	Post	Pre	Post	Pre	Post	Pre	Post
**Exacerbations^¶^**								
** 0**	4 (4)	44 (43)	2 (6)	15 (45)	0 (0)	16 (53)	2 (5)	13 (33)
** 1**	12 (11)	40 (39)	2 (6)	10 (30)	3 (10)	11 (37)	7 (18)	19 (47)
** 2**	40 (39)	17 (17)	14 (42)	7 (21)	12 (40)	3 (10)	14 (35)	7 (18)
** 3**	34 (33)	2 (2)	10 (30)	1 (3)	12 (40)	0 (0)	12 (30)	1 (2)
** 4**	11 (11)	0 (0)	4 (12)	0 (0)	3 (10)	0 (0)	4 (10)	0 (0)
** 5**	2 (2)	0 (0)	1 (3)	0 (0)	0 (0)	0 (0)	1 (2)	0 (0)
**AER**	2.88 (2.64–3.12)	0.96 (0.74–1.18)	2.75 (2.29–3.21)	0.96 (0.46–1.37)	3.33 (2.94–3.74)	0.71 (0.42–1.00)	2.67 (2.29–3.05)	1.15 (0.85–1.37)
**Relative reduction in AER from baseline, %**	66.7		64.9		78.8		56.6	

AER: annual exacerbation rate; EOS: eosinophils. ^#^: All pre–post comparisons in exacerbations amount and rate were different from baseline to 1-year follow-up with a p-value <0.01. ^¶^: Pre-treatment exacerbations were those that occurred before first tezepelumab dosage in the same time period as the follow-up time.

### Pulmonary function tests

**FIGURE 3:**
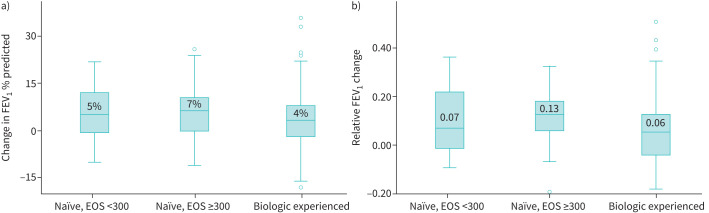
Changes in forced expiratory volume in 1 s (FEV_1_) at follow-up, as shown by a) total % predicted and b) as a relative change from baseline. EOS: eosinophils.

All patients had available follow-up pulmonary function test results. At follow-up, FEV_1_ % pred improved from a median (IQR) of 65 (49–82) % pred to 74 (55–88) % pred, p<0.01. An improvement in total FEV_1_ was also noted, from a median (IQR) of 1.69 (1.22–2.04) L to 1.9 (1.29–2.19) L, p<0.01. Changes in total FEV_1_ % pred and relative FEV_1_ are shown in figure 3. The largest improvements in FEV_1_ was seen in biologic-naïve patients with eosinophil counts >300 cells·μL^−1^, although differences between groups were not statistically significant. In total, 27% had an absolute increase of ≥10% pred in their FEV_1_ at follow-up and 51% had a relative increase in FEV_1_ of ≥10%. Among patients with available FEV_1_ in litres (n=79), 41% had an improvement of ≥100 ml. FEF_25–75%_ at baseline was a median (IQR) of 48 (26–79) % pred and at follow-up 60 (32–82) % pred, without a significant difference between them (p=0.092).

### Asthma-related treatments and symptoms

**FIGURE 4:**
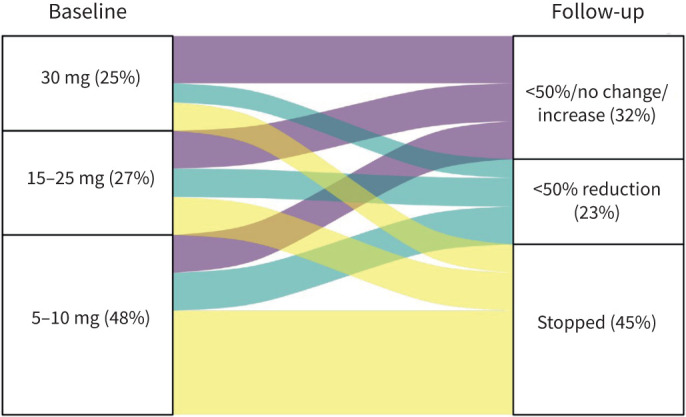
Maintenance oral corticosteroids doses (prednisone equivalent) at baseline and their changes at follow-up.

**FIGURE 5:**
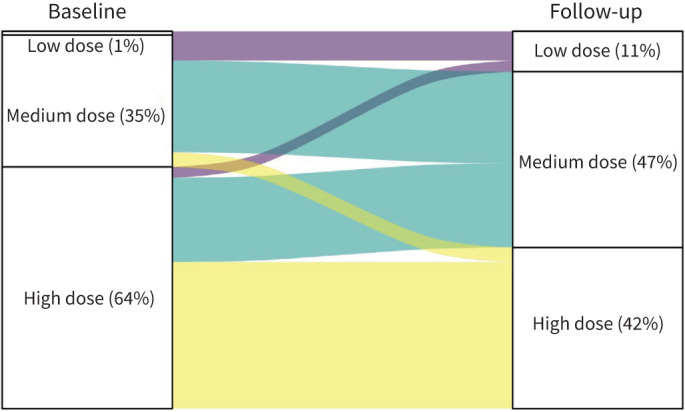
Inhaled corticosteroids doses at baseline and their changes at follow-up.

At baseline, 40 (39%) used mOCS. The mOCS doses (in prednisone equivalent) and changes in OCS at follow-up are shown in figure 4. At follow-up, 45% were no longer using OCS and an additional 23% had a reduction of ≥50% in their dose. ICS doses and changes at follow-up are shown in figure 5. At baseline, 64% were prescribed high-dose ICS, 35% medium-dose ICS and one patient low-dose ICS. There was a significant change in ICS doses from baseline to follow-up (p<0.01). Overall, 32% had a decrease in their level of ICS dose, while only 4% had an increase in ICS level during the 1-year follow-up. ACT scores were available for 76 patients. The median (IQR) ACT score at follow up was 19 (15–22) and 49% had an ACT score of 20 or above.

### Combined outcomes and predictors

26 patients (25%) had a combination of no exacerbations during follow-up and a relative increase of 0.1 or more in FEV_1_. Of those with baseline mOCS (n=40), 40% had a combination of no exacerbations during follow-up and mOCS cessation. Moreover, 23% had no exacerbations during follow-up, mOCS cessation and a relative increase of ≥10% in FEV_1_.

The rate of the combined positive outcome of no exacerbations and relative increase of ≥10% in FEV_1_ was higher in biologic-naïve patients with eosinophil counts >300 cells·μL^−1^ (*versus* all other patients: OR 2.81, 95% CI 1.11–7.15; p=0.03). No other associations were found between baseline variables and the mentioned outcomes, including sex, known allergy based on skin testing, IgE levels and nasal polyposis.

## Discussion

This multi-centre retrospective study provides real-world evidence on the use of tezepelumab therapy for severe asthma. To our knowledge, this study is one of the first studies describing real-world data beyond clinical trials for tezepelumab, specifically in a multicentre cohort. Our main findings include a major reduction in exacerbations regardless of peripheral eosinophil levels or prior biologic use. We also found improvements in FEV_1_ and reduction in mOCS. Even though our results were validated in all subgroups, having a peripheral eosinophil count of ≥300 cells·μL^−1^without prior biologic therapy was a predictor for improved response to treatment.

In Israel, tezepelumab can be considered for patients with severe asthma as a first-line biological treatment regardless of previous biological treatments. Therefore, unlike previous real-world studies on tezepelumab, which mostly included patients with prior biologic treatments [14], the majority of the patients in our cohort were biologic-naïve patients. In addition, the biologic drugs given to the patients in our cohort were almost exclusively anti-IL-5, anti-IL-5Rα or IL-4Rα, with only a few receiving the anti-IgE drug omalizumab, better reflecting the biologic treatments commonly used in current real-world practice.

The two phase-3 studies, PATHWAY and NAVIGATOR, confirmed a significant reduction in exacerbations following the initiation of tezepelumab (61.7–66%) regardless of eosinophilic phenotype. In our study, we observed a superior improvement in patients naïve to biological therapy with a high eosinophil count, which is consistent with the recent pooled analysis of the PATHWAY and NAVIGATOR studies [7, 9] and with another sub-analysis of these trials [8]. Although these and other studies have shown improved outcomes for patients with type-2 comorbidities, such as nasal polyps [15], we did not find similar associations, possibly due to the small sample size of patients with each comorbidity and the different patient population in our study, including those failing to achieve a good response to prior biologic therapy.

A significant exacerbation reduction rate of 63% was observed in a subgroup of patients with a lower cut-off of <150 cells·μL^−1^. This finding might be derived from tezepelumab's broad suppressive effect on AHR, suggesting that it is an effective choice for this subgroup of patients [16, 17]. Other biologics have also been shown to improve AHR [18, 19], but no head-to-head biologic studies have been adequately powered to assess AHR specifically, suggesting it could not solely explain the change in response and highlighting a need for further research. Our results add to previous real-world data showing comparable responses between eosinophilic *versus* noneosinophilic asthma [14]. Nevertheless, this subgroup of patients was underrepresented in previous RCTs and demonstrated a much lower exacerbation reduction rate [20].

The improvement in FEV_1_ in our study is also in line with the two main RCTs and with a recent meta-analysis [21], although some real-world data did not find a similar trend [22]. It is worth noting that in contrast to the RCTs, pulmonary function test improvements in our study were present in biologic-experienced patients as well as naïve patients.

Switching to tezepelumab after the failure of a different biologic therapy demonstrated significant effectiveness, with improvements in exacerbation rates. This switch was mostly due to a poor clinical response and failure to reduce asthma exacerbations. The add-on clinical effectiveness of tezepelumab is concurrent with several studies showing that in patients treated with eosinophil-targeting biologics, approximately half of the exacerbations were noneosinophilic, with low T2 biomarkers [23, 24]. Therefore, shifting to tezepelumab, which exerts beneficial pharmacological actions in both type 2 high and low asthma, could explain the high efficacy. Nevertheless, as mentioned above, this group had the lowest exacerbation reduction rate compared to biologic-naïve patients, regardless of eosinophil counts. In addition, the reductions in exacerbations among type-2 low patients in the NAVIGATOR trial were heterogeneous (reflected by the wide 95% confidence interval). These issues suggest that additional factors need to be considered, such as untreated or unstable comorbidities and different pathophysiological processes between groups.

The SOURCE study was a phase 3 trial that included patients with asthma on mOCS who were randomised to receive tezepelumab or placebo [25]. Interestingly, this study did not find a significant improvement in mOCS dose reduction. Since then, a reduction in mOCS was found by real-world studies [14, 26], supporting our findings. This discrepancy between clinical trial and real-world results may be influenced by study design, such as the lack of a placebo control group in the real-world studies and different study populations (*i.e*. more comorbidities in a real-world setting). Regarding ICS, at baseline, 64% of patients were prescribed high-dose ICS and 35% medium-dose ICS, and significant changes were noted in ICS treatment at the 1-year follow-up. This is supported by previous studies demonstrating safety in reducing ICS while on specific biological treatment, although clinical guidelines and valid clinical studies regarding ICS reduction while on tezepelumab are lacking [27].

We were unable to assess rates of clinical remission based on its accepted definition [28], due to several limitations of our study, as detailed below. Nevertheless, a combination of no exacerbation, steroid cessation/reduction and increased FEV_1_ were used as indicators for this important outcome, which occurred in 23% of relevant patients. In the NAVIGATOR phase 3 trial of tezepelumab, on-treatment clinical remission was achieved by 28.5%, although it was not significantly higher than the placebo [20]. High eosinophil count is a known predictor for remission with biologic therapy. Although tezepelumab has a proven efficacy for patients with low eosinophil counts, it was still shown by prior studies and ours to have a stronger effect in patients with higher counts [14, 20].

Our study has limitations. First, the retrospective design and lack of a control group precludes assessments of causality or a placebo effect, and limits control of possible confounders. Second, missing data on symptoms and patient perspectives limit our analysis and any conclusion on clinical remission. We also lacked details regarding the indications for treatment change in subjects with prior biological therapies and the compliance with ICS therapy that could obviously impact patient outcomes. Third, exhaled nitric oxide fraction, which has been previously reported as a predictor of tezepelumab clinical efficacy, was not routinely available and therefore was beyond the scope of our study. Finally, considering that tezepelumab has been reimbursed in Israel since 2023, an extended interval for real-world data on tezepelumab is still needed and further evaluations are warranted.

### Conclusion

Treatment of patients with severe asthma using tezepelumab for a median of 46 weeks was associated with overall improved asthma control, including significant reductions in asthma exacerbation rate, improved pulmonary function, cessation of mOCS, reduction in ICS dose and good symptom control. Our findings add to the scarce real-world data on tezepelumab for severe asthma. Tezepelumab was efficient in both biologic-naïve and biologic-experienced patients, and in patients with low and high eosinophil counts. By addressing all of the different patient sub-groups, we validate the role of tezepelumab across different asthma phenotypes, both as a first-line treatment and as a second-line option following failure of other biological treatments.

## Data Availability

All analyses made are given within the manuscript.
